# Operating Protocols of a Community Treatment Center for Isolation of Patients with Coronavirus Disease, South Korea

**DOI:** 10.3201/eid2610.201460

**Published:** 2020-10

**Authors:** EunKyo Kang, Sun Young Lee, Hyemin Jung, Min Sun Kim, Belong Cho, Yon Su Kim

**Affiliations:** Seoul National University Hospital, Seoul, South Korea

**Keywords:** respiratory infections, severe acute respiratory syndrome coronavirus 2, SARS-CoV-2, SARS, COVID-19, 2019 novel coronavirus disease, coronavirus disease, zoonoses, viruses, coronavirus, quarantine facility, community treatment center, CTC, South Korea

## Abstract

Most persons with confirmed coronavirus disease (COVID-19) have no or mild symptoms. During the COVID-19 pandemic, communities need efficient methods to monitor asymptomatic patients to reduce transmission. We describe the structure and operating protocols of a community treatment center (CTC) run by Seoul National University Hospital (SNUH) in South Korea. SNUH converted an existing facility into a CTC to isolate patients who had confirmed COVID-19 but mild or no symptoms. Patients reported self-measured vital signs and symptoms twice a day by using a smartphone application. Medical staff in a remote monitoring center at SNUH reviewed patient vital signs and provided video consultation to patients twice daily. The CTC required few medical staff to perform medical tests, monitor patients, and respond to emergencies. During March 5–26, 2020, we admitted and treated 113 patients at this center. CTCs could be an alternative to hospital admission for isolating patients and preventing community transmission.

Since the first suspected case was reported in December 2019 (*1*,*2*), the number of coronavirus disease (COVID-19) cases has risen steeply worldwide (*3*,*4*). In South Korea, COVID-19 outbreaks occurred at religious facilities and the number of cases increased drastically, especially in Daegu City and the North Gyeongsang Province, and the number of patients with asymptomatic or mild symptoms increased exponentially (*5*,*6*). In the early stages of the COVID-19 epidemic, all patients with diagnosed COVID-19 were hospitalized in negative-pressure isolation units to treat the disease and prevent the spread of infection. However, because the infection spread rapidly, the number of patients exceeded the number of available negative-pressure isolation beds. Because of limited medical resources and the COVID-19 epidemic curve, concerns grew that new facilities would be needed to isolate and care for patients in South Korea.

The National Health Insurance System (NHIS) of South Korea offers complete access to healthcare for the entire population (*13*). South Korea’s medical utilization rate is the highest, 16.6 outpatient visits per capita per year, among Organization for Economic Cooperation and Development (OECD) countries (*14*). Citizens of South Korea have high access to medical services. Before the COVID-19 pandemic, no one in the country anticipated a situation in which hospital admission would be denied. South Korea has 2.6 times more hospital beds than other OECD countries, 12.3/1,000 population. However, the country only has 1,027 negative-pressure isolation beds, and these are not distributed across all regions. When the COVID-19 pandemic reached South Korea, the number of available negative-pressure isolation beds decreased, and patients could not be admitted to the hospital because of the shortage of medical facilities, especially in regions where outbreaks mainly occurred.

When an imbalance between the demand and supply of medical resources exists, adequate triage of patients is critical for allocating limited resources to patients who can benefit the most (*7*). In a large-scale study from China, Wu et al. (*8*) suggested that ≈80% of COVID-19 symptomatic patients were reported to have mild upper respiratory infection without hypoxia, and only 20% of infected patients needed medical services. Until March 25, 2020, the crude mortality rate in South Korea was 1.4%, and estimates suggested the severity of COVID-19 in the country would not be high (*9*). However, considering asymptomatic carrier transmission (*10*), the high reproductive number (R_0_ = 2.2) (*11*), and the possibility of sudden deterioration (*12*), even patients with mild or no symptoms should be isolated and monitored.

On March 2, 2020, the government of South Korea started operating community treatment centers (CTCs) to provide quarantine, regular examination, and monitoring for asymptomatic and mildly symptomatic patients with laboratory-confirmed COVID-19. By March 25, a total of 17 CTCs were serving patients with mild symptoms nationwide. The CTC is designed to monitor and isolate patients with mild conditions during emerging infectious disease outbreaks. We describe the structure and operating protocols of a CTC operated by Seoul National University Hospital (SNUH) during the COVID-19 pandemic. 

## Materials and Methods

### Study Setting in the SNUH-CTC

SNUH is a teaching hospital with 1,700 beds. The Mungyeong Human Resource Development (HRD) Center, 153 km from SNUH, is a 7-story, 100-room facility with accommodations that is normally used for training SNUH staff. SNUH converted Mungyeong HRD to a CTC in cooperation with the government’s CTC operating policy and established a monitoring center inside SNUH. SNUH-CTC began operating on March 5, 2020 as the third CTC in the country.

### Admission and Discharge Criteria

#### Screening Criteria for Patients for CTC

The Korea Centers for Disease Control and Prevention (KCDC) classified the severity of COVID-19 into very severe, severe, mild, and asymptomatic (*15*) (Appendix Table 1). Mild COVID-19 is defined as alert and meeting >1 of the following conditions: <50 years old, >1 underlying conditions, and temperature <38°C with antipyretic drugs. Asymptomatic is defined as a patient who is alert, <50 years old, has no underlying disease, is a nonsmoker, and has a temperature of <37.5°C without antipyretic drugs. Patients classified as severe or very severe were admitted to hospitals; CTCs only accepted patients classified as having mild or asymptomatic COVID-19.

Patients with mild COVID-19 met >1 of the following criteria for CTC admission: they did not necessarily require hospitalization; they only required monitoring; they were unable to properly self-isolate (for instance, they had no suitable place to live or lived with persons in a high-risk group); or, as determined by local government, they needed to be admitted to a CTC. Medical staff assessed patients and excluded persons at high-risk for deterioration from CTCs and recommended hospitalization.

#### Criteria for Discharge from the CTC

KCDC has 2 criteria for releasing patients from quarantine. Symptomatic patients can be discharged if symptoms disappear and they have negative results on 2 reverse-transcription PCR (RT-PCR) tests >24 hours apart. KCDC recommended PCR amplification of the viral E gene as a screening test and amplification of the RdRp region of the open reading frame 1b gene as a confirmatory test. RT-PCR is considered positive only when all the genes are detected, based on the opinions of experts who detected weak and nonspecific amplification in the clinical specimens of patients who received negative results. Asymptomatic patients can be discharged if they have 2 negative RT-PCR tests >24 hours apart within 7 days of diagnosis.

We conducted our study in accordance with the World Health Association’s Declaration of Helsinki (https://www.wma.net). The study was approved by the institutional review board of SNUH (IRB no. H-2003-163-1112).

## Results

### Overall Structure

**Figure 1:**
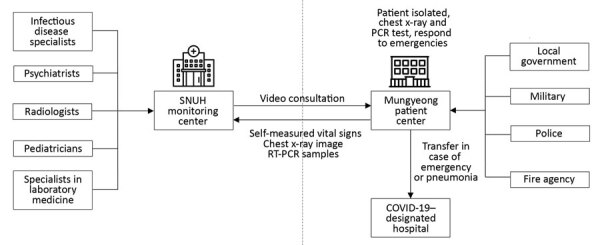
Overall structure of the SNUH community treatment center (SNUH-CTC) for isolating mildly symptomatic or asymptomatic patients with coronavirus disease, South Korea. SNUH-CTC was divided into a monitoring center at SNUH in Seoul and a patient center 153 km away in Mungyeong. Boxes indicate various agencies and organizations the provided staff to help run SNUH-CTC and support operations. Arrows indicate direction of information, services, or patient transport. RT-PCR, reverse transcription PCR; SNUH, Seoul National University Hospital.

SNUH-CTC consisted of 2 centers: the patient center in Mungyeong HRD, where patients were admitted, and the monitoring center in Seoul at SNUH, where medical staff provided video consultation services (Figure 1). In the patient center, personnel from the Ministry of Health and Welfare, local government, hospitals, military, police, and fire agencies stayed and provided various services necessary for the operation of the CTC (Appendix Table 2).

### Patient Center 

**Figure 2:**
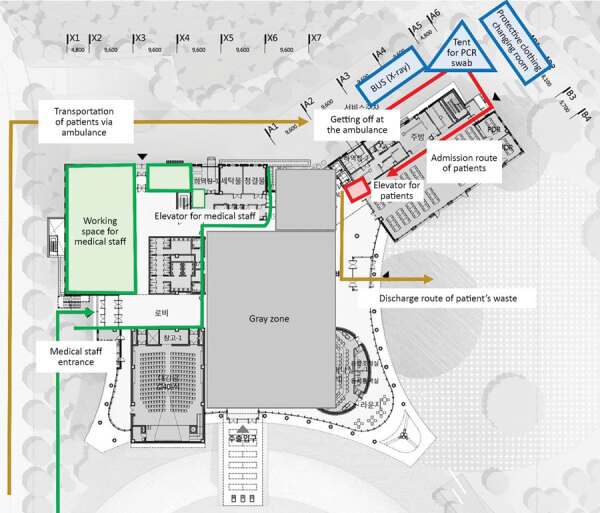
Diagram of the patient center of Seoul National University Hospital community treatment center (SNUH-CTC) located in the Mungyeong Human Resource Development (HRD) Center, Mungyeong, South Korea. CTCs were set up to isolate and monitor mildly symptomatic or asymptomatic patients with coronavirus disease. Green indicates the clean areas in which staff worked. Red indicates contaminated areas in which patients stayed. Gray zone indicates areas in which staff performed other activities, such as collecting patient’s samples or removing waste. Yellow indicates routes for patient admission and removal of patient waste. Blue indicates external services kept outside of the building.

We divided the Mungyeong HRD Center into a clean area, in which medical and operating staff worked, and a contaminated area, where patients lived. Each area had a designated entrance separate from the other. We designated an area between the clean and contaminated areas as a gray zone in which personnel could remove personal protective gear or perform other required activities, such as collecting patient samples or removing waste (Figure 2).

Mungyeong HRD Center had internet service and SNUH installed an additional network to access the hospital’s electronic medical record (EMR) system. Supplies for conducting RT-PCR tests, such as swabs and a refrigerator, and a mobile radiography bus were placed next to the building. According to the national guidelines for COVID-19, physicians collected RT-PCR samples on a 2-day cycle for negative cases and on a 3- or 7-day cycle for positive cases and sent the samples to SNUH to be tested.

To detect pneumonia early, patients with abnormal findings on chest imaging had daily chest radiographs until normalization; patients without abnormal findings had chest radiographs every 3 days. Radiographs were read by a radiologist in SNUH through the picture archiving and communication system. RT-PCR and radiographic results could be checked in the Mungyeong HRD and the SNUH monitoring center through the EMR system. Essential medicines, such as antipyretic drugs and cough medicines, were stored in the Mungyeong HRD and provided by a physician’s prescription.

A physician or nurse was always on duty in the Mungyeong HRD patient center to respond to emergencies. The physicians came from many specialties, including emergency medicine, family medicine, and general surgery, to address various patient conditions and emergencies. During the day, 2 physicians and 2 nurses were on duty; at night 1 physician and 2 nurses were on duty. During each shift, 1 physician acted as the medical director, and 1 nurse acted as an infection manager. All staff, including medical staff, were checked twice a day for fever and respiratory symptoms, such as cough, sputum, stuffy nose, sore throat, chest discomfort, and dyspnea. When staff reported symptoms, the medical director checked the staff member and provided a RT-PCR test if necessary.

Each room of the Mungyeong HRD patient center was equipped with an automatic blood pressure monitor, digital thermometer, and pulse oximeter so that patients could check vital signs independently. Meals were provided three times a day, and laundry was done by patients in their rooms. Most patients were not permitted to have visitors, but children could be visited by parents or guardians.

### Monitoring Center 

The monitoring center at SNUH was equipped with computers and monitors, smartphone devices, webcams, headsets for video consultation, and 2 large dashboard monitors to check the patients’ vital signs and symptoms. Patients admitted to the CTC checked their blood pressure, body temperature, pulse, respiratory rate, and oxygen saturation at 9 am and 4:30 pm each day. Patients reported their symptoms, including respiratory symptoms, twice a day through a questionnaire sent through a smartphone application, and the nurse on duty monitored the responses and vital signs. The nurse provided video consultations twice a day from 9 am–12 pm and 5–8 pm. If the nurse decided that video consultation with a doctor was necessary, the doctor provided additional consultation. The doctor regularly monitored patients’ vital signs and symptoms once a day and conducted regular video consultations once every 2 days. On average, nurses and doctors provided video consultations for ≈5 minutes per patient per consult and monitored patients’ symptoms and vital signs for ≈3 minutes per patient monitoring session (Appendix Table 3). 

Radiologists at SNUH read and provided results for patients’ chest radiographs. When patients had abnormal radiography findings or the patient’s symptoms worsened, the physician at Mungyeong HRD Center consulted with an infectious disease specialist at SNUH.

Patients in the CTC underwent a comprehensive psychiatric assessment once a week to evaluate for depressive mood, anxiety, risk for suicide, and posttraumatic stress. The questionnaire included a standard depression module, a generalized anxiety disorder assessment, suicidality screening, a posttraumatic stress disorder checklist, and somatic symptom assessment. For high-risk groups, psychiatrists conducted a separate in-depth psychological consultation by using the video consultation system. 

The video consultation model for patients in isolation with diagnosed COVID-19 integrated an interprofessional clinical team to provide patient-centered care. By reducing direct face-to-face consultations with infectious patients, we helped ensure the safety of medical staff. Video consultation was essential for providing patient care and helped integrate services, including monitoring vital signs and patient symptoms; providing consultation with nurses, physicians, infectious disease specialists, and radiologists; and in-depth psychological consultation by a psychiatrist, when needed. 

### Preparation for Emergencies

The CTC established an emergency referral system with nearby medical institutions to respond to emergencies or increased symptoms. In an emergency, medical staff on duty in the CTC donned protective gear to visit the patient’s room. The patient center was equipped with an emergency cart normally used in the hospital, a portable oxygen tank, and a stretcher with a negative-pressure air tent for transferring patients to the ambulance area, if needed.

**Figure 3:**
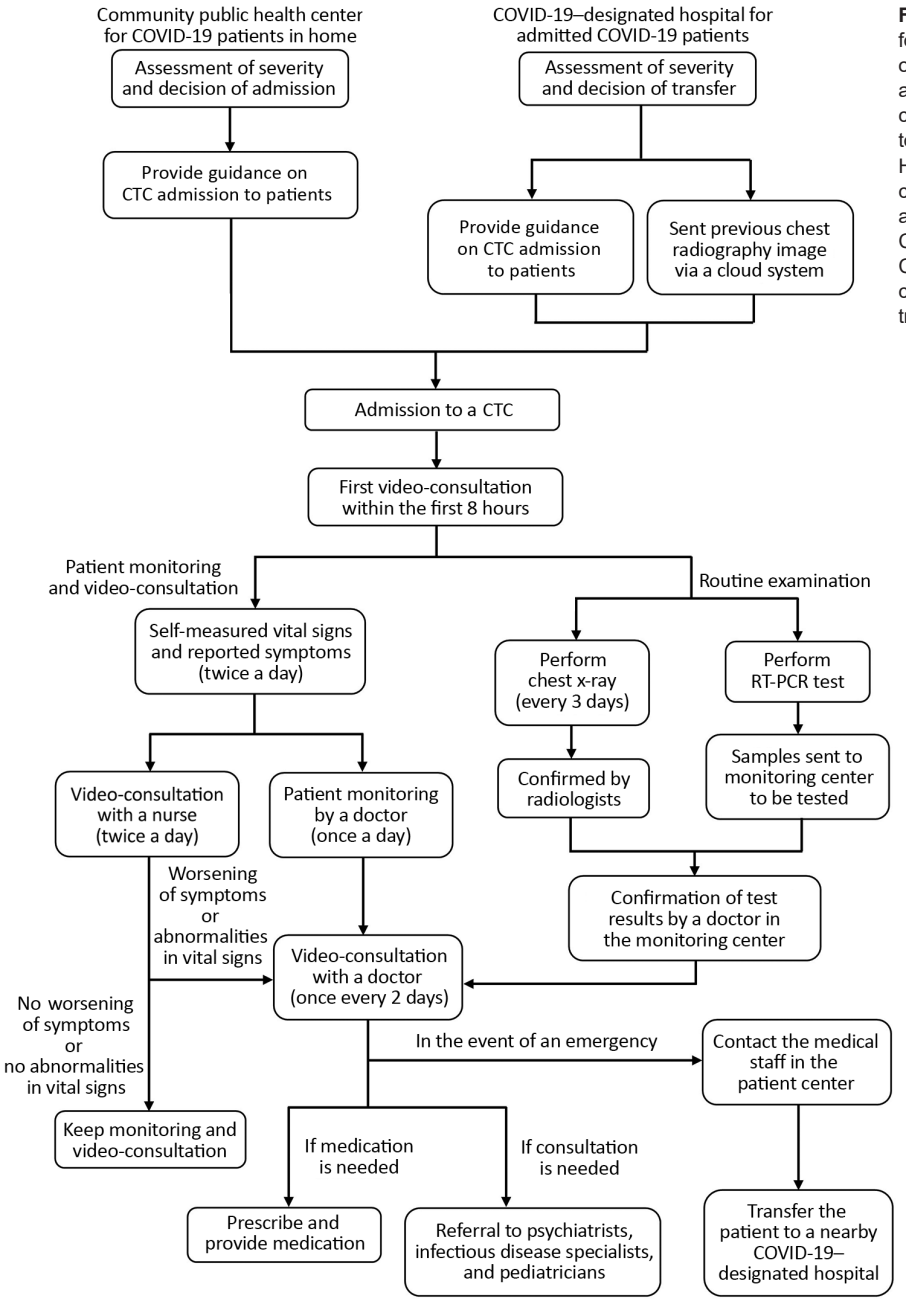
Flow chart of protocols for admission and management of mildly symptomatic or asymptomatic patients with coronavirus disease admitted to the Seoul National University Hospital community treatment center (SNUH-CTC) for isolation and monitoring, South Korea. COVID-19, coronavirus disease; CTC, community treatment center; RT-PCR, reverse transcription PCR.

Patients requiring hospitalization were transferred to a hospital with a negative-pressure isolation unit that KCDC designated for treating COVID-19 patients. Criteria for transport to a hospital included abnormal vital signs measured every day for >3 days or evidence of pneumonia on chest radiographs. Patients were transferred by ambulance from the nearest ambulance station. If an emergency occurred, such as abrupt respiratory failure, the patient was first transferred to the nearest emergency department for treatment and stabilization before being transferred to a hospital bed (Figure 3).

### Characteristics of Patients in the SNUH-CTC

In total, 113 patients were admitted to the SNUH-CTC during March 5–26, 2020. Among patients, 59 (52.2%) were female and, 54 (47.8%) were male, the average age was 30.4 years (range 9–65 years), and 7 (6.2%) had underlying conditions, 4 of whom had hypertension. The average number of days of illness before admission to the CTC was 5.1 days. Among patients admitted with symptoms, 31 (27.4%) had cough, and 1 (0.9%) had fever. Four (3.5%) patients developed fever within 3 days after admission. Twelve (10.6%) patients had abnormalities in chest radiographs performed on the day of admission, but most were nonspecific haziness or opacity; only 1 patient appeared to have pneumonia (Table 1).

**Table 1:** Characteristics of 113 patients with mild or asymptomatic coronavirus disease admitted to the Seoul National University Hospital community treatment center for isolation and monitoring, South Korea

Characteristics	Value
Sex	
M	54 (47.8)
F	59 (52.2)
Age, y (mean + SD)	30.4 ± 12.9
Average length of illness, d (mean + SD)	5.1 ± 3.5
Fever	
At admission	1 (0.9)
<3 d	4 (3.5)
<2 weeks	15 (13.3)
Never	98 (86.7)
Symptoms at admission	
Cough	31 (27.4)
Sputum	25 (22.1)
Rhinorrhea	18 (15.9)
Chest discomfort	8 (7.1)
Sore throat	7 (6.2)
Dyspnea	5 (4.4)
Underlying conditions	
Hypertension	4 (3.5)
Diabetes	1 (0.9)
Asthma	1 (0.9)
Chronic bronchitis	1 (0.9)
None	106 (93.8)
Vital signs, mean (SD)	
Systolic blood pressure, mm Hg	113.6 (11.8)
Diastolic blood pressure, mm Hg	75.9 (9.2)
Respiratory rate, times/min	16.6 (5.4)
Heart rate, bpm	82.6 (11.5)
Body temperature, °C	36.3 (0.6)
Oxygen saturation, %	96.1 (4.5)
Chest radiograph†	
Abnormal	12 (10.6)
Within normal limits	101 (89.4)

*Values are no. (%) except as indicated. †At admission to the community treatment center.

### Outcomes of Patients in the SNUH-CTC 

During March 5–26, the SNUH-CTC admitted 113 patients; 103 were admitted directly from home, and 10 were transferred from the hospital during the recovery period. During the 3 weeks studied, 49 patients recovered and were discharged, and 2 patients were transferred to a COVID-19–designated facility for hospitalization (Table 2). The average length of stay in the CTC was 15.7 days (interquartile range [IQR] 5–21 days), and the average interval from diagnosis to discharge was 19.5 days (IQR 10–27 days). One patient was transferred to a hospital after persistent pneumonia on chest radiographs for 3 days, and another patient was transferred for close monitoring because dyspnea developed and the patient needed oxygen at 1 L/min. In both cases, the medical staff staying in the CTC evaluated the patients in their rooms and decided to transfer them after detecting the deterioration on consultation. Both patients were safely admitted to the hospital. 

**Table 2:** Admission and discharge of patients in the Seoul National University Hospital community treatment center, South Korea

Category	No. (%)	Mean days (interquartile range)		
		Quarantine time before admission	Length of stay	From diagnosis to discharge
Admission				
From home	103 (91.2)	5.8 (3–5)		
From hospital	10 (8.8)	11.6 (6–16.8)		
Discharge method				
Recovered	49 (43.4)		15.7 (5–21)	19.5 (10–27)
Transferred	2 (1.8)			
Not discharged	62 (54.8)			

## Discussion

South Korea established CTCs for isolation and monitoring of patients with no or mild symptoms of COVID-19 during a pandemic in which the demand for medical resources have exceeded the supply. SNUH converted an existing accommodation facility into a medical facility and provided video consultation via a smartphone to minimize staff contact with infectious patients. The hospital operated the CTC and provided medical services and public officials from the Ministry of Health and Welfare, local government, military, police, and the fire agency supported the operation by providing food delivery and patient transfer. During the 3-week operation, 113 asymptomatic and mildly symptomatic patients were admitted to SNUH-CTC for monitoring and care.

As COVID-19 spreads worldwide, the shortage of medical resources has become a serious problem in many countries (*16*,*17*). The lack of medical resources, such as hospital beds, intensive care units, and ventilators, can hinder the ability to treat patients adequately (*18*,*19*). In addition, during the pandemic crisis, a shortage of quarantine facilities, in this case hospitals, could increase the transmission of infectious diseases (*20*). When the demand for medical resources is greater than the supply, proper patient triage and resource allocation are crucial. During this pandemic, hospitals might not have sufficient medical equipment, such as ventilators and extracorporeal membrane oxygenation, for all patients. To maximize resources and save the most patients, hospitals with sufficient medical equipment can provide medical services for critically ill patients. However, carefully monitoring the disease progress, even for mild conditions, can assist in documenting clinical course of emerging infectious diseases. In addition, appropriate isolation of patients who test positive for COVID-19 can prevent further spread of disease (*20*).

South Korea has many acute care beds and high medical accessibility with the NHIS (*21*). Despite an 80% ratio of asymptomatic and mildly symptomatic patients in the early stages of the epidemic, all COVID-19 patients in South Korea were admitted to negative-pressure isolation rooms according to the principle of first come, first served (*8*). As the pandemic rapidly progressed, hospital beds became scarce (*22*), and >2,000 patients waited at home for hospital admission, including patients in high-risk groups, such as persons >65 years of age and those with underlying conditions (*23*). Before CTCs were opened in South Korea, at least 2 patients died at home waiting for hospital admission and the need for medical facilities and redistribution of medical resources increased (*22*,*24*). Some patients hospitalized in the early stages of the endemic did not need active treatment but were required to be isolated and monitored. However, infected patients who were already hospitalized could not be discharged because of the possibility of sudden deterioration and difficulties in the control and monitoring during self-isolation at home (*20*). 

A new quarantine model is needed to ensure beds in fully equipped hospitals for severe disease cases and the capacity to monitor and isolate asymptomatic and mildly symptomatic patients. For the current COVID-19 pandemic, South Korea implemented CTCs as an intermediate model between self-isolation at home and hospital isolation. The core aim of CTCs is to isolate patients in single rooms with bathrooms and provide care with telemedicine. Because the CTC model can be adapted as surge capacity in various types of facilities, such as resorts and hotels, CTCs could quickly secure a quarantine bed in a pandemic crisis. We found that CTCs can be an alternative to fully functioning hospitals and home isolation. CTCs enabled the country to preserve hospital resources for the sickest patients and isolate patients from the community to prevent further transmission. In addition, CTCs provided an opportunity for physicians to observe COVID-19 disease progression and triage patients who deteriorate to higher care, instead of leaving patients at home.

We found that allowing patients to independently measure their vital signs and providing telemedicine consultations had several advantages. First, reduced contact between healthcare workers and patients minimized the risk for infection for healthcare workers. During the pandemic, the infection or quarantine of healthcare workers will exacerbate the problem of already scarce medical resources (*16*). Second, because telemedicine is possible regardless of distance, CTCs would enable regions with sufficient resources to support regions with insufficient resources. In our model, the CTC and the monitoring centers were >100 km apart. The SNUH-CTC used a video consultation model instead of conventional telephone interviews because we could observe additional visual signs or diagnostic clues through video conferences (*25*,*26*). By using self-measurement equipment and advanced telecommunication technology, including smartphones, we were able to maximize these services.

South Korea opened its first CTC on March 2; by March 26, a total of 3,292 patients were admitted to 17 CTCs, representing 35.6% of the 9,241 cumulative confirmed COVID-19 cases in the country. During those 24 days, no deaths or instances of respiratory failure were reported in the 17 CTCs operated. The CTC model offers safe monitoring and isolation for asymptomatic or mildly symptomatic patients with diagnosed COVID-19 during the pandemic. During shortages of medical resources, appropriate triage of patients and allocation of resources are needed so that critically ill patients receive the highest level of care and patients with less severe infection can be safely monitored and treated. The CTC model also could be useful during natural disasters in which the demand for medical care overwhelms the supply.

We note a few limitations of CTCs. First, because a CTC is not a hospital, appropriate response to emergencies, such as respiratory failure, might be difficult. During our CTC operations, we chose to transfer patients to surrounding COVID-19–designated hospitals for emergency treatment; future planning should include hospitals with emergency services within a short distance of the CTC. Second, because we were not able to observe patients in real time, we might not have detected a sudden emergency. To protect patient privacy, we did not install a closed-circuit television in patient rooms, but we trained the patients to contact the medical staff immediately if they had a medical emergency. However, other countermeasures, such as patient alarm bells in each room, might be needed. Third, the CTC also is a quarantine facility; patient discomfort and depression might increase during long-term admission. To try to assure patients’ mental health, we provided various psychiatric interventions; in addition to other medical services, mental health should be built into further isolation and quarantine models. Fourth, our CTC did not have a negative-pressure isolation function as an infectious disease facility.

In conclusion, to safely isolate and monitor the asymptomatic and mildly symptomatic patients with COVID-19, South Korea developed the CTC model as an intermediate between hospitalization and self-isolation at home. By classifying patients according to the disease severity and underlying conditions, asymptomatic and mildly symptomatic patients can be safely monitored and treated at CTCs.

Appendix Additional information on operating protocols of Seoul National University Hospital community treatment center where mildly symptomatic or asymptomatic patients with coronavirus disease were isolated and monitored.
