# Supplementary material for: Operating Protocols of a Community Treatment Center for Isolation of Patients with Coronavirus Disease, South Korea
Source: Emerg Infect Dis. 2020 Oct;26(10):2329–37. doi: 10.3201/eid2610.201460 (PMC7510742; doi:10.3201/eid2610.201460)
Supplement: Appendix — Additional information on operating protocols of Seoul National University Hospital community treatment center where mildly symptomatic or asymptomatic patients with coronavirus disease were isolated and monitored. [file 20-1460-Techapp-s1.pdf]

Article DOI: https://doi.org/10.3201/eid2610.201460

# Operating Protocols of a Community Treatment Center for Isolation of Patients with Coronavirus Disease, South Korea

## Appendix

**Appendix Table 1.** Stage classification and definition of coronavirus disease conditions defined by the Korea Centers for Disease Control and Prevention

| Classification | Description |
|---|---|
| Asymptomatic | Alert, <50 years of age, no underlying disease, nonsmoker, and body temperature <37.5°C without antipyretic drugs |
| Mild | Alert and meeting ≥1 of the following conditions: <50 y of age, ≥1 underlying conditions, or body temperature <38°C with antipyretic drugs |
| Severe | Alert and meeting ≥1 of the following conditions: body temperature >38°C with antipyretic drugs or dyspnea |
| Very severe | Not alert |

**Appendix Table 2.** Role and services provided by organizations to the Seoul National University Hospital community treatment center*

| Organization | Role and service |
|---|---|
| Ministry of Health and Welfare | Contact point for Central Disaster and Safety Countermeasure Headquarters, provision and application of guidelines for CTC |
| Local government | Support for goods, such as personal protective equipment, and administrative support, such as providing meals, disinfection services, and waste disposal |
| Hospital (SNUH) | General operation of the CTC and providing medical services, including patient monitoring and assessment and prescribing medications |
| Mungyeong HRD center | Facility management |
| Military | Food delivery, patient movement control |
| Police | Outside access control, patrol around facility |
| Fire agency | Contact point for emergency services for transfer, and patient movement control |

*CTC, community treatment center; HRD, human resources and development; SNUH, Seoul National University Hospital.

**Appendix Table 3.** Video consultation care model in the Seoul National University Hospital community treatment center*

| | No. or duration/day/medical professional | |
|---|---|---|
| Service | Doctor | Nurse |
| Video consultation | 1× every 2 d | 2×/day |
| Monitoring patients' symptoms and vital signs | 1×/day | 2×/day |
| Average time per video consultation | 5 min | 5 min |
| Average number of patients per medical staff | 30 | 18 |
| Total workforce required for 113 patients | 3 or 4 doctors | 12 nurses |

*The center housed 113 patients during March 5–26, 2020.
